# A Longitudinal Study on the Influence of Various Activities in a Teacher Training University on Self-Esteem and Self-Efficacy in the Teaching Profession and on Career Aspirations to Become a Teacher

**DOI:** 10.1192/j.eurpsy.2026.11498

**Published:** 2026-07-31

**Authors:** K. Uchida, K. Yamasaki

**Affiliations:** Department of Psychology and Educational Science, Naruto University of Education, Naruto, Tokushima, Japan

## Abstract

**Introduction:**

In recent years in Japan, issues concerning children’s health and adjustment have been reported, and teachers’ working conditions have also deteriorated. As a result, sick leave and burnout among teachers have increased. Moreover, fewer university students are choosing teaching as their future career. Yamasaki and Uchida (2025) suggested that a preventive approach is necessary for university students in teacher training programs.

**Objectives:**

This study aimed to longitudinally examine the influence of various activities in a teacher training university on self-esteem and self-efficacy in the teaching profession and on career aspirations to become teachers.

**Methods:**

Participants were 193 university students (*M* = 18.43 years, *SD* = 0.54) who aimed to become teachers. They completed a battery of six questionnaires at two time points: once at university entry (Time 1) and again in their second year (Time 2). The questionnaires included the Scale of Self-Esteem in the Teaching Profession (Yamasaki & Uchida, 2023); the Scale of Self-Efficacy in the Teaching Profession (Yamasaki & Uchida, 2024), which assesses classroom and instructional management, student guidance, collaboration with colleagues, and overall self-efficacy; the General Self-Efficacy Scale (Yamasaki et al., submitted manuscript); the Teaching Career Aspiration Scale; and the Reasons for Changes in Teaching Career Aspiration Scale, which assessed whether teaching career aspiration had increased or decreased and the reasons for these changes. The reasons were assessed at Time 2, reflecting the past year.

**Results:**

We calculated the changes in each variable from Time 1 to Time 2. Pearson correlation coefficients were then computed to examine the relationships between these changes and the reported reasons for increases or decreases in teaching career aspiration. The results indicated that “having conducted satisfactory lessons at the university” was positively correlated with self-esteem and self-efficacy (student guidance) in the teaching profession as a reason for an increase. In contrast, “loss of interest in teaching due to coursework related to teacher education” was positively correlated with self-esteem in the teaching profession as a reason for a decrease.

**Conclusions:**

This study suggests that self-esteem and self-efficacy in the teaching profession are associated with the reasons for both increases and decreases in teaching career aspiration. Especially, the findings indicate that university coursework may help prevent a decline in teaching career aspiration. These results can be considered by university staff to maintain or enhance students’ teaching career aspiration.

**Disclosure of Interest:**

None Declared

